# Cost Analysis of Medical versus Surgical Management of Glaucoma in Nigeria

**Published:** 2010-10

**Authors:** Afekhide E Omoti, Omolabake T Edema, Benedicta A Akpe, Patricia Musa

**Affiliations:** University of Benin Teaching Hospital, Benin City, Nigeria

**Keywords:** Glaucoma, Health Care Costs

## Abstract

**Purpose:**

To analyze the cost of glaucoma medical therapy and compare it with that of surgical management in Nigeria.

**Methods:**

The cost of glaucoma drugs and that of surgical therapy in patients who attended the eye clinic of the University of Benin Teaching Hospital, Benin City, Nigeria, between December 2002 and November 2008 were calculated over a 3 year period of follow-up. Costs of medical and surgical therapy were compared based on November 2008 estimates.

**Results:**

One hundred and eight patients met the inclusion criteria of the study, of which, 90 patients (83.33%) received medical therapy and 18 patients (16.67%) underwent surgery. The most expensive drugs were the prostaglandin analogues, travoprost (Travatan) and latanoprost (Xalatan). The least expensive topical drugs were beta-blockers and miotics. The mean annual cost of medical treatment was US$ 273.47±174.42 (range, $41.54 to $729.23) while the mean annual cost of surgical treatment was US$ 283.78±202.95 (range, $61.33 to $592.63). There was no significant difference between the mean costs of medical and surgical therapy over the 3-year period (P = 0.37). Older age (P = 0.02) and advanced glaucoma (P < 0.001) were associated with higher costs of therapy.

**Conclusion:**

The cost of medical therapy was comparable to that of surgical therapy for glaucoma in Nigeria over a 3-year period.

## INTRODUCTION

Glaucoma is the second leading cause of blindness worldwide and is estimated to have affected 60.5 million people in 2010, increasing to 79.6 million by 2020.^1^ In glaucoma, the optic nerve is progressively damaged, causing defects in the visual field, usually asymptomatic until central vision is affected.^2^,^3^ Unfortunately, by the time characteristic visual field defects are demonstrable, one third or more of the optic nerve fibers are already damaged; by the time patients complain of significant field loss, the remaining axons are probably 10% or fewer.^4^ The purpose of glaucoma treatment is to preserve visual function while minimizing adverse effects of therapy, thereby enhancing patient health and quality of life.^2^,^5^,^6^ Although trabeculectomy is regarded as the standard form of treatment for patients with primary open angle glaucoma in developing countries including Nigeria,^7^ many glaucoma patients are treated medically. This is especially true in teaching hospitals and other tertiary centers which are concentrated in urban areas, partly because of patients’ unwillingness to undergo surgery (since there will be no improvement in vision) and partly due to fear of surgery.^8^

Primary open angle glaucoma (POAG) requires lifelong monitoring; this places an increasing financial strain on the health service system as the elderly population increases. The volume and cost of glaucoma drugs have been shown to have increased dramatically in both Northern Ireland and the Republic of Ireland from 1996 to 2003, probably as a result of a combination of changing demographics and a changing approach towards the management of patients with glaucoma and ocular hypertension.^9^ It has been demonstrated in Europe, that resource utilization and direct medical costs of glaucoma management increase with worsening disease severity.^10^ This has also been shown in the United States.^11^ However, there is a paucity of information on glaucoma therapy costs in Sub-Saharan Africa where patients frequently present with advanced stages of the disease.^12^ The purpose of this study is to analyze the cost of glaucoma drugs and compare it with that of surgical therapy in Benin City, Nigeria.

## METHODS

Data was obtained from the medical records of all patients diagnosed with POAG and normal tension glaucoma, who attended the eye clinic of the University of Benin Teaching Hospital, Benin City, Nigeria, between December 2002 and November 2008. The patients were included in the study if they had at least 3 years of continuous follow up. Only patients who were compliant with therapy were included in the study. Patients were included in the surgery group if they had primary trabeculectomy or had a short period of medical therapy, not exceeding one month, before trabeculectomy. Patients were excluded if they had less than 3 years of continuous follow up, concomitant ocular diseases likely to affect glaucoma treatment-related resource consumption, or were subsequently enrolled at any time in a clinical trial. Patients who were not compliant with therapy were also excluded.

Collected data included patient demographics, number of ophthalmology clinic visits, number and type of glaucoma medications and surgeries, perioperative drugs in subjects who had undergone glaucoma surgery, and any postoperative procedure. All clinical tests documented in the files were also recorded. Glaucomatous optic disc appearance was categorized using the modified grading by Jay^13^ as follows:

- Stage 1: Suspicious shape of the optic nerve, but cup/disc ratio less than or equal to 0.6.
- Stage 2: Pathological: cup/disc ratio greater than 0.6, but less than 0.9.
- Stage 3: End stage: cup/disc ratio greater than or equal to 0.9.

The hospital pharmacy, the accounting department, and four nearby pharmacies were consulted to provide available unit costs for surgical procedures and medications extracted from the case records. The direct annual cost of treatment per person-year, including a breakdown of costs attributed to glaucoma surgeries, cataract extractions, and glaucoma medications, was calculated for all patients. The costs were measured in Nigerian Naira (N) and expressed in United States Dollars ($). The exchange rate during the period of the study was N 130.00 to $1.00. The costs of drugs or surgery used for the study were based on estimates in November 2008.

A 5 ml bottle of an ocular medication prescribed twice daily or its equivalent (for example, a 2.5 ml bottle of a medication prescribed once daily) was assumed to represent a one-month supply for any given patient. Data on medication usage were collected at every visit and patients were assumed to adhere fully to medical regimens unless otherwise noted in the case records. The cost of therapy was calculated from the perspective of patients and only direct costs of drugs and surgery were considered. Only out-of-pocket payments were considered as none of the subjects had insurance policies.

Socio-economic status was classified using the patient’s occupation as modified from the British Registrar General’s Classification.^14^ Class I comprises higher professions, such as medical doctors, engineers, businessmen, etc. Class II includes lesser professions such as nurses, teachers, etc. Class III consists of skilled workers such as typists, clerks, technicians, etc. Class IV comprises semi-skilled workers such as petty traders, machine operators, etc. Class V includes unskilled workers such as subsistence farmers, housewives, etc.

### Data Analysis

In order to calculate the number of resources consumed per person-time, the total number of each consumed resource was added and then divided by the total number of follow-up visits. For patients who underwent surgery, the cost of surgery, hospital stay, and preoperative, intraoperative and postoperative drugs were calculated as the total cost of surgery. If the patient needed additional drugs to control intraocular pressure (IOP) or additional surgery for complications, these were also added to the cost of trabeculectomy in the surgery group. Data obtained from this study were analyzed using the Instat GraphPad version 2.05a software (GraphPad Software Inc., La Jolla, CA, USA). Mean and standard deviations (SD) of the costs of drugs and surgery were compared using the student t-test. 95% confidence intervals (95%CI) and the standard error of mean (SEM) were also determined. P-values less than 0.05 were considered significant. Factors affecting the cost of glaucoma therapy were determined using the student t-test and the one way analysis of variance (ANOVA) where appropriate.

## RESULTS

**Figure 1:**
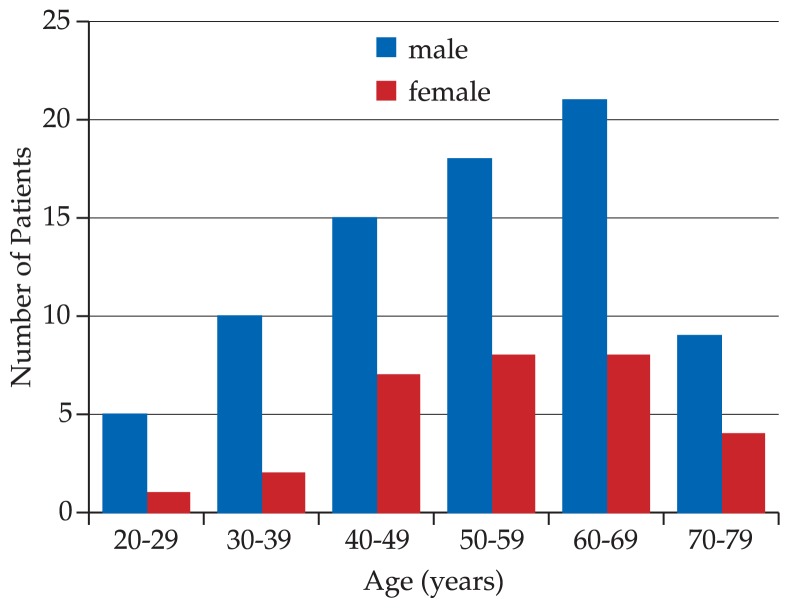
Age and sex distribution of glaucoma patients.

Overall, 308 new patients who attended the eye clinic during the period of the study were diagnosed with POAG or normal tension glaucoma. Of these, 108 subjects (35.06%) met the inclusion criteria which included 78 male (72.22%) and 30 female (27.78%) patients with mean age of 58.14±10.8 (range 22 to 76) years. The age and sex distribution of study subjects is shown in figure 1. Sixteen patients (14.81%) were illiterate, 6 (5.56%) had primary education, 26 (24.07%) had secondary education, and 60 (55.56%) had tertiary education. Socio-economic status of the patients is shown in table 1. Ninety patients (83.33%) received medical therapy and 18 patients (16.67%) underwent surgery.

The cost of commonly available drugs in Benin City, Nigeria is shown in table 2. Most subjects used topical preparations but some were on acetazolamide tablets. The presented price of acetazolamide was the cost of one month of therapy with one tablet three times daily.

The mean annual cost of medical therapy was US$ 273.47±174.42, SEM = $ 31.85, median = $ 261.35, and 95% CI = $ 208.35–338.60 (range, $ 41.54 to $ 729.23). The mean monthly cost of medical therapy was $ 22.78±14.53, SEM = $ 2.65, median = $ 21.78, and 95% CI= $ 17.21–28.21 (range, $ 3.46 to $ 60.77). The mean daily cost of medical treatment was $ 0.75±0.48, SEM = $ 0.09, median = $ 0.72, and 95% CI = $ 0.57–0.93 (range, $ 0.11 to $ 2.00).

The lowest annual cost of medical therapy was associated with monotherapy with timolol maleate 0.5% drops, while the highest annual cost of medical therapy was related to combination therapy with latanoprost 0.005% (Xalatan) or travoprost (Travatan), dorzolamide (Trusopt) or brinzolamide (Azopt), and timolol maleate 0.5% (Timoptol).

The cost of trabeculectomy in the University of Benin Teaching Hospital was $ 103.85. The cost of perioperative drugs was $ 46.31, and that for an average 4-day hospital stay was $ 33.85. Thus, the total cost of trabeculectomy per eye was $ 184.00.

The mean annual cost of surgical treatment was US$ 283.78±202.95, median = $ 253.92, and 95% CI = $ 70.76–496.79 (range, $ 61.33 to $ 592.63). The mean monthly cost of surgical treatment was $ 23.65±16.91, median = $ 21.16, and 95% CI = $ 5.90–41.40 (range, $ 6.11 to $ 49.38). The mean daily cost of surgical treatment was $ 0.79±0.56, median = $ 0.71, and 95% CI = $ 0.20–1.38 (range, $0.17 to 1.65).

Patients with the lowest annual cost of surgical therapy were those who had successful trabeculectomy without additional need for medical therapy for IOP control. Only 3 of 18 patients (16.67%) had complete success with trabeculectomy and did not require additional medical treatment. Patients with the highest annual cost of surgical therapy were those who had unsuccessful trabeculectomy with additional need for medical therapy for IOP control and cataract surgery.

There was no significant difference between the mean cost of medical and surgical therapy (t=1.134, df=2, P=0.37).

The cost of medical therapy according to the number of prescribed medications is demonstrated in table 3, which shows that cost rises as the number of medications increases. The cost of surgical therapy is shown in table 4, which shows that the cost of surgical therapy was highest in patients who developed complications and those who required reoperations or additional cataract surgery. Older age and advanced stages of glaucoma were significantly associated with higher costs of therapy (Table 5).

## DISCUSSION

Large-scale, prospective, randomized multicenter trials have provided evidence that lowering IOP in glaucoma can arrest the progression to blindness.^15^,^16^ Glaucoma is most commonly treated with topical medications in developed countries; surgery is usually performed if medications fails to lower IOP adequately.^6^ Glaucoma drugs are commonly divided into six main groups: prostaglandin analogues, β-blockers, miotics, sympathomimetics, carbonic anhydrase inhibitors, and combination drugs. For more than 20 years, topical β-blockers dominated the market, overtaking the older miotics and sympathomimetics in volume of sales.^9^ However, in the past decade a number of new drugs have been introduced, which have had a profound effect on the management of glaucoma.^6^,^9^

The majority of patients in the present study were managed medically which is contrary to what is expected based on reports that trabeculectomy is the standard form of treatment for patients with primary open angle glaucoma in developing countries.^7^ This may reflect a change in the choice of therapy, probably because of the range of effective drugs now available, and the poor acceptability of glaucoma surgery in Benin City, Nigeria.^8^ A low proportion of surgical management has also been reported from Western Nigeria, where only 8.2% of a series of glaucoma patients had trabeculectomy.^17^ Although the majority of our patients had tertiary education and belonged to social classes I and II, the annual income in Nigeria is much less than that of developed countries. Subjects with tertiary education were those who had post-secondary education such as university, polytechnic, or other professional education. The minimum wage in Nigeria is only N 5,500.00 ($42.31) monthly and N 66,000.00 ($507.69) per annum.^18^ This amount is just slightly higher than the price of latanoprost or travoprost. These figures imply that a patient on minimum wage cannot afford a combination therapy which includes prostaglandin analogues, even if he/she spends all his/her income. The average patient will also have to save all of his/her monthly income for 5 months to afford trabeculectomy in one eye. Even patients in higher professions still have to spend a significant amount of their earnings to be able to pay for combination therapy or surgery.

The most expensive drugs were the prostaglandin analogues, travoprost (Travatan) and latanoprost (Xalatan). Other expensive drugs included topical carbonic anhydrase inhibitors, dorzolamide and brinzolamide. Although other drugs, such as brimonidine (Alphagan), are available in Benin City, none of the patients in this series used them. These drugs were usually not prescribed because they were not commonly available. The least expensive topical drugs were β-blockers and miotics. The oral carbonic anhydrase inhibitor, acetazolamide, was also inexpensive. Although acetazolamide is not indicated for long term use, it was commonly prescribed because of its efficacy in the short term and because poor patients could afford a few tablets at a time, thus reducing non-compliance due to the cost of drugs.

There was no significant difference in the mean annual, monthly, or daily costs of medical versus surgical treatment. The average cost of surgical therapy was however, slightly higher over a 3-year period. This is in contrast to what is expected, since surgical patients are expected to pay once for their procedure while patients on medical therapy continue to purchase drugs indefinitely. Eventually, medical therapy should become more expensive. Unfortunately, many glaucoma patients who had undergone trabeculectomy needed additional medical therapy for IOP control and may also have incurred extra costs for management of postoperative complications such as cataracts, infection, bleb-related complications, etc. Trabeculectomy tends to fail over time because of fibroblastic proliferation and subconjunctival fibrosis which occurs during the process of wound healing.^19^ Adjunctive use of antifibrotic agents such as mitomycin C at the site of surgery has significantly reduced the risk of bleb failure.^20^ Sherwood et al^21^ have argued that it is wrong to believe that a one-step surgery will save money. Their 1991 estimates show that the cost of bilateral uncomplicated glaucoma surgery roughly equals 8 years of medical treatment. Since about one third of the patients may need additional medical therapy or reoperations for failed surgery, premature cataract extraction, and other complications, the final cost of surgery may be even more. In the current study, patients who had complications and required additional procedures had the highest cost of surgical therapy.

The lowest cost of medical therapy was observed for monotherapy with timolol maleate 0.5% which was slightly lower than that of successful and uncomplicated trabeculectomy not requiring additional medical therapy. However if these two groups of patients had been followed up for up to 5 years, the mean monthly cost of surgery ($3.06) would have been lower than the mean monthly cost of medical therapy ($3.46). If they were followed up for even longer, the difference would become more significant. Unfortunately, most of our glaucoma patients are usually lost to follow-up after a few years and many African patients will eventually have poorly controlled IOP after surgery because of the propensity for fibrosis. These patients will need additional medical therapy, revision of trabeculectomy, or repeat surgery, thus increasing the cost of surgical therapy.

Older age and late stages of glaucoma were significantly associated with higher costs of glaucoma therapy. This is because patients who present with advanced disease are treated more vigorously with more medications and surgery. In these patients the target IOP is set lower because optic nerve damage is severe. Similar findings have been reported from Europe^10^ and the United States^11^. Older patients are more likely to present later and with advanced disease.

Although our study groups were non-homogenous, this study was designed to look back and measure what it cost for the average patient to undergo medical therapy or surgery, and compare them. The annual cost of surgery was used rather than the onset surgery cost and maintenance cost, in order to correct the assumption that surgery is a one-off event, when in reality, many cases require additional medical therapy. Therefore, the average cost of surgery should also include the cost for management of complications, as well as any additional therapy that may be required to control IOP.

One limitation of this study is its retrospective nature. Furthermore, patients were not randomized to medical versus surgical therapy. Thus, the sample size was lopsided such that the majority of the patients were in the medical group and only a few underwent surgery. The therapeutic protocol was determined mainly by the level of IOP and the severity of the disease, as well as patients’ choice and ability of the patient to afford the prescribed treatment. Also, because the study is retrospective, details of indirect costs could not be elicited. The study thus focused on direct costs of surgery and drugs. The confounding role of age, disease severity, and socio-economic status was not taken into consideration. In the original study population from which the study sample was selected, the majority of patients were lost to follow up before 3 years. Poor follow up in glaucoma patients had been reported in other studies from Nigeria.^7^

In conclusion, the mean cost of medical therapy was comparable to that of surgical therapy over a 3-year period. The prostaglandin analogues were the most expensive drugs, while β-blockers and miotics were the most affordable. Older age and advanced glaucoma were associated with higher costs of glaucoma therapy. The minimum wage in Nigeria can barely cover the cost of newer antiglaucoma drugs or surgery.

## Figures and Tables

**Table 1:** Socioeconomic status of glaucoma patients

Social class		Number of patients
I.	Higher professions	38 (35.19%)
II.	Lesser professions	39 (36.11%)
III.	Skilled workers	19 (17.59%)
IV.	Semi-skilled	4 (3.70%)
V.	Unskilled	8 (7.41%)

Total		108 (100%)

**Table 2:** Commonly available antiglaucoma drugs and their unit cost

Drug (volume)	Trade name	Manufacturer	Unit cost (N)	Unit cost (US$)
Timolol (5ml)	Timolol 0.5%	Hovid	400.00	3.08
Timolol (5ml)	Timolol 0.5%	Martindale	400.00	3.08
Timolol (5ml)	Nyolol 0.5%	Novartis	900.00	6.92
Timolol (5ml)	Optimol 0.5%	Ashford	450.00	3.46
Timolol (5ml)	Cusimolol 0.5%	Alcon	650.00	4.62
Timolol (5ml)	Timoptol 0.5%	Merck, Sharpe & Dohme-Chibret	1,100.00	8.46
Betaxolol (5ml)	Betoptic 0.5%	Alcon	900.00	6.92
Pilocarpine (10ml)	Pilocarpine 2%, 4%	Martindale	450.00	3.46
Pilocarpine (10ml)	Pilocarpine 2%, 4%	Drugfield	450.00	3.46
Pilocarpine (10ml)	Pilocarpine 2%, 4%	Cusi	500.00	3.85
Dorzolamide (5ml)	Trusopt 2%	Merck, Sharpe & Dohme	3,450.00	26.54
Brinzolamide (5ml)	Azopt	Alcon	2,800.00	21.54
Latanoprost (2.5ml)	Xalatan 0.005%	Pfizer	4,200.00	32.31
Latanoprost (2.5ml)	Xalost 0.005%	Taejoon Pharm	3,300.00	25.38
Latanoprost (2.5ml)	Prostan 0.005%	ShanDong LuNan Better	2,700.00	20.77
Travoprost (2.5ml)	Travatan 40μg/ml	Alcon	4,500.00	34.62
Acetazolamide	Acetomid (250mg tablets)	Hovid	900.00	6.92

**Table 3:** Cost of glaucoma medications over 3 years

No. of glaucoma medications	No. of patients	Mean annual cost (US$)	Mean monthly cost (US$)	Mean daily cost (US$)
1	15 (16.67%)	$ 158.22	$ 13.18	$ 0.44
2	54 (60.00%)	$ 312.55	$ 26.05	$ 0.88
3	17 (18.89%)	$ 393.42	$ 32.78	$ 1.09
4	4 (4.44%)	$ 553.85	$ 46.15	$ 1.54

**Table 4:** Cost of surgical therapy over 3 years

Type of surgery	No. of patients	Mean annual cost (US$)	Mean monthly cost (US$)	Mean daily cost (US$)
Trabeculectomy only	3 (16.67%)	$ 61.33	$ 5.11	$ 0.17
Trabeculectomy plus drugs for less than 1 year	3 (16.67%)	$ 296.72	$ 24.73	$ 0.82
Trabeculectomy plus drugs for more than 1 year	2 (11.11%)	$ 218.26	$ 18.19	$ 0.61
Trabeculectomy plus drugs for more than 2 years	1 (5.56%)	$ 139.79	$ 11.65	$ 0.39
Trabeculectomy with postoperative complications and/or repeat surgery	6 (33.33%)	$ 408.72	$ 34.06	$ 1.14

**Table 5:** Factors affecting cost of therapy

Factors	Frequency	Mean annual cost ± SD (US$)	Test	df	P-value
Age					
< 40 years	18 (16.67%)	$ 249.00 ± 49.54	F=4.034	107	0.0205

40–60 years	48 (44.44%)	$ 278.31 ± 58.12			

> 60 years	42 (38.89%)	$ 294.31 ± 58.05			

Sex					
Male	78 (72.22%)	$ 280.62 ± 58.98	t=0.6351	106	0.5267

Female	30 (27.78%)	$ 272.69 ± 55.56			

Socioeconomic status					
Higher professions	38 (35.19%)	$ 279.75 ± 58.88	F=0.05572	107	0.9941

Lesser professions	39 (36.11%)	$ 282.56 ± 58.18			

Skilled workers	19 (17.59%)	$ 286.40 ± 52.95			

Semi-skilled	4 (3.70%)	$ 275.92 ± 50.58			

Unskilled	8 (7.41%)	$ 282.87 ± 53.72			

Stage of glaucoma					
Stage 1	42 (38.89%)	$ 257.38 ± 56.40	F=8.188	107	0.0005

Stage 2	35 (32.41%)	$ 272.65 ± 54.10			

Stage 3	31 (28.70%)	$ 309.69 ± 54.86			

df, degree of freedom
